# Bridging the Gap: Nurses’ Integral Role in Oral Cancer Prevention and Management

**DOI:** 10.7759/cureus.57379

**Published:** 2024-04-01

**Authors:** Ribwar A Mohammed, Kochr A Mahmood, Sirwan K Ahmed

**Affiliations:** 1 College of Nursing, University of Raparin, Rania, IRQ; 2 Faculty of Medicine, Koya University, Koya, IRQ

**Keywords:** patient education, palliative care, oral cancer management, oral cancer screening, nurses

## Abstract

This editorial explores the important role that nurses play in bridging the gap in the prevention and management of oral cancer. Despite advances in medical science, oral cancer remains a significant global public health challenge, with high mortality rates due to late diagnosis and inadequate treatment. Nurses, with their multifaceted contributions, are indispensable in addressing this challenge. The editorial emphasizes the key role of nurses in patient education and awareness, early detection and screening, comprehensive patient care, supportive services, and advocacy. Nurses are frontline educators, advocates, caregivers, and collaborators in the fight against oral cancer. Through their expertise, compassion, and dedication, nurses have a significant impact on patient outcomes, reducing mortality rates, and promoting a healthier future for those affected by oral cancer. Recognizing and supporting the crucial role of nurses in the management of oral cancer is essential for advancing prevention, detection, and treatment efforts.

## Editorial

The global prevalence of oral cancer is a major public health concern as it continues to increase worldwide. According to the World Health Organization (WHO), oral cancer ranks as the eleventh most common form of cancer. The worldwide estimated incidence of oral cancer rose from 354,864 cases as reported by GLOBOCAN in 2018 to 377,173 cases as reported by GLOBOCAN in 2020, yet the number of new fatalities stayed constant at around 177,000 [1].

Despite advancements in medical research, the mortality rate associated with oral cancer remains alarmingly high, primarily due to delayed diagnoses and inadequate management strategies [1]. Nurses play a vital role in bridging the gap between prevention efforts, early detection, and effective oral cancer management [2]. This editorial aims to explore the significance of nurses in oral cancer prevention and management, focusing on their contributions to patient care, education, and advocacy.

Nurses are at the forefront of patient education and proactive awareness campaigns [3]. They empower individuals by providing comprehensive knowledge about oral cancer risk factors, symptoms, and prevention. Through community outreach programs, health fairs, and one-on-one engagements, nurses educate individuals about the importance of routine oral screenings, tobacco cessation, adopting a healthy lifestyle, and the need for early detection. By equipping individuals with this crucial information, nurses help reduce the incidence of oral cancer and promote early diagnosis, improving treatment outcomes.

Early detection is essential for effectively managing oral cancer and improving survival rates. Nurses play a crucial role in facilitating early detection through routine screenings and comprehensive assessments [3]. In healthcare settings, nurses thoroughly examine patients' oral health during regular check-ups, identifying suspicious lesions or abnormalities that require further investigation. Additionally, nurses collaborate with dentists, oncologists, and other healthcare professionals to develop and implement screening programs for high-risk populations. Their expertise in recognizing early signs of oral cancer enhances patient prognosis and overall quality of life. By recognizing and addressing the importance of nurses in oral cancer management, we can ensure more effective prevention strategies, early detection, and improved patient outcomes. Nurses are essential in closing the gap and making a significant impact on the management of this devastating disease. Nurses provide holistic care to individuals diagnosed with oral cancer, addressing their physical, emotional, psychological, and social well-being. Throughout the entire journey, from diagnosis to treatment and survivorship, nurses serve as advocates, educators, and compassionate caregivers, offering unwavering support at every stage. They help patients understand treatment options, manage side effects, and cope with the emotional burden of the disease. They also collaborate with multidisciplinary teams to create personalized care plans, ensuring continuous and effective care for each patient.

In cases where a cure is not possible, nurses play a crucial role in providing supportive and palliative care to those with advanced oral cancer [4]. Their focus is on alleviating pain, managing symptoms, and improving the quality of life for patients and their families. This includes empathetic listening, spiritual support, and counseling to help individuals navigate the challenges of terminal illness and end-of-life care. Through their presence and commitment, nurses ensure that patients receive dignified and compassionate care throughout their cancer journey.

Nurses are also vital advocates for evidence-based standards and initiatives to improve oral cancer prevention, detection, and treatment [5]. They work with professional organizations, government agencies, and community stakeholders to advocate for legislation supporting tobacco control measures, access to oral healthcare services, and funding for cancer research. Additionally, nurses engage in public awareness campaigns, media outreach, and educational programs to raise awareness about oral cancer and promote policy changes that prioritize cancer prevention and control. By using their voices and expertise, nurses drive meaningful change locally, nationally, and internationally in the fight against oral cancer.

In conclusion, nurses play a crucial role in bridging gaps in oral cancer management. They do this through patient education, early detection, comprehensive care, supportive services, and advocacy. As frontline healthcare providers, nurses have a unique position to make a significant impact on preventing, detecting, and treating oral cancer. By using their expertise, compassion, and dedication, nurses are essential in improving patient outcomes, reducing mortality rates, and promoting a healthier future for those affected by oral cancer. It is important to recognize and support the vital role of nurses in oral cancer management. This means ensuring they have the necessary resources and recognition to continue their life-saving work.
